# Structural similarity of loops in protein families: toward the understanding of protein evolution

**DOI:** 10.1186/1471-2148-5-10

**Published:** 2005-02-03

**Authors:** Anna R Panchenko, Thomas Madej

**Affiliations:** 1 Computational Biology Branch, National Center for Biotechnology Information, National Institutes of Health, Bethesda, MD 20894, USA

## Abstract

**Background:**

Protein evolution and protein classification are usually inferred by comparing protein cores in their conserved aligned parts. Structurally aligned protein regions are separated by less conserved loop regions, where sequence and structure locally deviate from each other and do not superimpose well.

**Results:**

Our results indicate that even longer protein loops can not be viewed as "random coils" and for the majority of protein families in our test set there exists a linear correlation between the measures of sequence similarity and loop structural similarity. Results suggest that distance matrices derived from the loop (dis)similarity measure may produce in some cases more reliable cluster trees compared to the distance matrices based on the conventional measures of sequence and structural (dis)similarity.

**Conclusions:**

We show that by considering "dissimilar" loop regions rather than only conserved core regions it is possible to improve our understanding of protein evolution.

## Background

Globular proteins are considered to be structurally similar if their regular secondary structure elements can be superimposed well and are connected in the same order. The loop regions connecting secondary structures demonstrate less regularity in their conformations even though short loops linking specific secondary structures can be classified into distinct classes [1-6]. The structures and sequences in loop regions may deviate from each other so that they do not superimpose well and as a result loops are very often not aligned by structure-structure or sequence alignment methods. Loops apparently do not contribute much to protein stability but may be quite important for protein specific function and for the interaction with other components of the cell. In our previous work we showed that a measure derived from the loop regions can distinguish homologous from analogous proteins with the same or higher accuracy compared to the conventional measures which are based on comparing proteins in structurally aligned regions only [7].

Recently it has been observed that structural variation in the core of homologous proteins is linearly correlated with sequence changes [8,9]. As was also shown several years ago, the probability of insertion and deletion events, which occur predominantly in the loop regions, strongly depends on the evolutionary distance between two homologous proteins [10,11]. Based on these observations one might argue that more closely related proteins may exhibit more similarity in the structure of their loop regions compared to distantly related proteins and the structural loop (dis)similarity should correlate with evolutionary distance.

To check this hypothesis we performed an analysis of structural variation in the loop regions within different homologous protein families using a recently introduced new measure of loop similarity [7]. This new measure is based on the concept of the Hausdorff metric, which is used in mathematical topology to define a distance between two point sets of a metric space. It does not require an alignment or one to one correspondence between two point sets. We show that there exists a linear correlation between the average structural change in the loop regions and the evolutionary distance, which allows us to use the loop (dis)similarity measure for inferring the phylogenetic history of homologous protein families.

## Methods

### Test set

To select sets of homologous proteins the Conserved Domain Database (CDD) version 1.62 was taken, which can be accessed at [12]. The CDD collection of protein domain alignments included curated CDDs [13] and preprocessed domain families imported from SMART and PFAM, altogether 6222 protein domain families[14]. Upon import, the sequences from SMART/PFAM alignments with more than 75% identity with known structures were substituted by the most similar structures from the Protein Data Bank [15].

Each CDD family was decomposed into a set of pairwise structure-structure alignments. Structural alignments were computed by the VAST algorithm [16] and only those structures which had more than 80% mutual overlap between the VAST alignment footprint and CDD footprint were considered in the analysis. The footprint for a given sequence was defined as a region between the first and the last residues aligned by VAST or CDD. Those families containing short sequence repeats and having average alignment length less than 50 residues were excluded from the test set. The structural pairs within the remaining CDD families were disregarded if at least one of the following conditions held true:

- at least one structure in a pair had X-ray resolution of greater than 3.0 Å

- the Blast E-value calculated for the VAST alignment exceeded 0.01

- at least one structure in a pair contained a chain discontinuous domain inconsistently aligned between VAST and CDD

- at least one structure in a pair contained more than 25% of its nonaligned loops with missing residues.

To ensure that protein families span a wide range of sequence similarity, all families were examined and those having less than 30% sequence identity span were not considered in further analysis. The redundancy between protein families was checked by using the procedure implemented in the CDART algorithm [17] and not more than 2 protein families from the same CDD cluster were retained in the final test set. At the end, the test set comprised 59 CDD families with more than 10 structurally aligned pairs of homologs. This test set covered a wide range of functional and structural classes and the list of test families together with their length, number of protein pairs and correlation coefficients is shown in Table 1.

**Table 1:** List of the names of 59 test protein families together with their CDD accession names, lengths, number of protein pairs, Pearson correlation coefficients between LHM (AHM) and normalized Blast bitscore. The families are ordered with respect to decreasing quality of LHM correlation. The supplementary table is available at [27].

***Family name***	***CDD acc***	***Length***	***#Obs***	***AHM***	***LHM***
Xylose_isom	pfam00259	381	28	-0.99	-0.98
MHC_I	pfam00129	175	28	-0.95	-0.96
PTPc	smart00194	248	25	-0.92	-0.96
IPT	smart00429	97	21	-0.90	-0.94
ZnMc_1	smart00235	137	34	-0.83	-0.94
RNAse_Pc	cd00163	99	25	-0.82	-0.94
gpdh_C	pfam02800	153	39	-0.72	-0.93
Aamy_C	smart00632	81	31	-0.94	-0.90
peroxidase	pfam00141	240	48	-0.90	-0.90
copper-bind	pfam00127	81	87	-0.84	-0.89
CBM_20	pfam00686	94	15	-0.91	-0.89
RnaseA	pfam00074	98	44	-0.48	-0.87
IGv	cd00099	105	133	-0.78	-0.86
ADH_zinc_N	pfam00107	337	64	-0.93	-0.86
ldh_C	pfam02866	143	29	-0.93	-0.86
RIP	pfam00161	232	28	-0.87	-0.85
Peptidase_C1	pfam00112	200	55	-0.82	-0.85
ZnMc_2	cd00203	134	23	-0.87	-0.85
PROF	cd00148	120	15	-0.90	-0.85
plant_peroxidase	cd00314	236	76	-0.90	-0.83
alpha-amylase_C	pfam02806	78	39	-0.93	-0.82
sodcu	pfam00080	139	15	-0.98	-0.81
fer2_1	cd00207	78	38	-0.86	-0.80
Pept_C1	smart00645	202	90	-0.86	-0.79
ferritin	pfam00210	152	19	-0.94	-0.79
ldh	pfam00056	135	44	-0.82	-0.78
SH2	pfam00017	86	21	-0.48	-0.78
flavodoxin	pfam00258	143	26	-0.88	-0.78
EFh	cd00051	57	59	-0.75	-0.77
rhv_1	cd00205	195	71	-0.86	-0.76
LYZ1_1	smart00263	116	67	-0.66	-0.75
aldo_ket_red	pfam00248	277	28	-0.93	-0.73
COesterase	pfam00135	485	28	-0.80	-0.72
TIG	pfam01833	89	39	-0.90	-0.72
fer2_2	pfam00111	69	73	-0.77	-0.70
beta-lactamase	pfam00144	264	45	-0.90	-0.70
rhv_2	pfam00073	216	95	-0.86	-0.70
GLECT	cd00070	124	28	-0.80	-0.67
globin	pfam00042	133	96	-0.74	-0.66
GST_C	pfam00043	107	77	-0.77	-0.63
LYZ1_2	cd00119	109	24	-0.43	-0.61
PA2c	smart00085	102	210	-0.29	-0.57
lipocalin	pfam00061	131	55	-0.62	-0.56
phoslip	pfam00068	102	102	-0.21	-0.54
proteasome	pfam00227	189	56	-0.80	-0.51
UBCc	smart00212	141	45	-0.79	-0.50
Sm	smart00651	63	30	-0.54	-0.49
Tryp_SPc	smart00020	208	561	-0.55	-0.46
CLECT_1	smart00034	90	35	-0.59	-0.44
crystall	pfam00030	81	10	-0.76	-0.41
CLECT_2	cd00037	93	263	-0.45	-0.36
RHO	smart00174	173	10	-0.52	-0.36
IGc1	cd00098	88	85	-0.65	-0.32
Tryp_SPc	cd00190	211	378	-0.55	-0.31
MHC_II_beta	pfam00969	86	32	-0.52	-0.26
ADK	pfam00406	174	28	-0.37	-0.19
Rho	cd00157	172	66	-0.20	-0.16
Phycobilisome	pfam00502	148	15	-0.85	-0.10
ADF	smart00102	116	10	-0.85	0.34

### Measures of structural and sequence similarity

To measure the sequence similarity between homologous proteins from the same family we used a Blast bitscore normalized by the alignment length. Among structure similarity measures used in this paper, two of them, RMSD and alignment-based Hausdorff measure (AHM) were computed by comparing the proteins in structurally aligned regions, while the loop-based Hausdorff measure (LHM) quantified the difference in the loop regions.

The root mean squared deviation (RMSD) was calculated using the superposition algorithm due to McLachlan [18]. The AHM and LHM measures were based on the mathematical concept of Hausdorff distance[19]. Let *A *= {*a*_1_,..., *a*_*m*_} and *B *= {*b*_1_,..., *b*_*n*_} be finite point sets in a Euclidean space. The Hausdorff distance between the sets *A *and *B *is then defined by:

*d*_*H *_(*A*, *B*) = max {min _*j *_*d*(*a*_1_, *b*_*j*_),..., min _*j *_*d*(*a*_*m*_, *b*_*j*_), min _*i *_*d*(*a*_*i*_, *b*_1_),..., min _*i *_*d*(*a*_*i*_, *b*_*n*_)}     (1)

Here the terms *d*(*a*_*i*_, *b*_*j*_) denote the usual Euclidean distance between the points. In other words, the Hausdorff distance between the sets *A *and *B *is the smallest distance such that every point *a*_*i *_∈ *A *is within this distance of some point *b*_*j *_∈ *B *and vice versa. Hausdorff distance can be calculated under the assumption that the *C*α atoms for both structures are in a common coordinate frame which is defined by the structural alignment between two domains. The Hausdorff measure for loops (LHM) was calculated as an average of Hausdorff distances over all loops in the protein pair, where *n*_*s *_is the number of aligned secondary structure elements:



The "loop" was defined as a region between two consecutive aligned secondary structure elements and:

*h*_*i *_= 0, if the *i*-th loop regions do not have any unaligned residues;

*h*_*i *_= *d*_*H *_(*A*_*i*_, *B*_*i*_), where *A*_*i *_contains the set of *C*α coordinates of non-aligned residues in the *i*-th loop of the first structure in a pair, the last aligned residue from the preceding aligned region and the first aligned residue from the following aligned region. Similarly, *B*_*i *_is defined for the second structure in a pair. The sets (*A*_*i*_, *B*_*i*_) are defined to include two aligned residues so that the measure can be defined even if one of the sets of non-aligned residues is empty. The Hausdorff measure for the structurally aligned regions (AHM) was defined similarly. In this case, instead of the sets that contain the coordinates for the *C*α atoms in the loops, we use the coordinates for the *C*α atoms in the aligned segments and average over the number of aligned segments.

The correlation analysis between the measures of sequence and structural similarity, linear/nonlinear regression analyses and cluster analysis were performed using Splus version 6. Pearson (ρ) and Spearman correlation coefficients were calculated to quantify the accuracy of linear correlation. The P-value under the null hypothesis that the correlation coefficient between two variables is equal to zero has been estimated and those families with the P-values less than 0.01 were considered as having statistically significant correlation. The cluster analysis was done using the complete linkage clustering [20] where the distance between two clusters was measured as a maximum distance between a point in one cluster and a point in another cluster. The cluster trees based on p-distance and LHM were compared using the Phylip program [21] by generating 1000 bootstrap alignments from the structural alignments of a protein family and by calculating p-distance based cluster trees from the bootstrap alignments. The bootstrap support for the LHM based tree or different partitions of this tree was calculated by counting how many times the LHM topology occurs among the bootstrap cluster trees.

## Results and discussion

Tables  1 and 2 show the accuracy of correlation obtained between the various measures of structural similarity (RMSD, AHM and LHM). As can be seen from these tables, the correlation quantified by the Pearson correlation coefficient is quite high for most of the families and half of the families have coefficients between -0.76 and -0.81 depending on the structural similarity measure used (Spearman rank correlation coefficients were shown to be very close to those reported in Tables 1 and 2). This result is consistent with the studies of Wood and Pearson who showed on a smaller test set of 35 protein families that half of them have correlation coefficients greater than 0.878 [8]. In their case the sequence-structure correlation was quantified, however, by using only the measures based on the structurally aligned regions of the proteins.

The dependence of structural similarity on sequence similarity in some cases can be more accurately described by the nonlinear regression model taking into account higher order quadratic terms. To quantify how much the nonlinear terms improve the data fitting, we use the ratio of squared correlation coefficient for linear () and nonlinear () models (). In the overall test set only 12 families have *r*^2 ^– ratio smaller than 0.9 (with LHM used as a structural similarity measure) indicating that for these cases adding the non-linear term improves the performance of modeling by about 10%.

**Figure 1:**
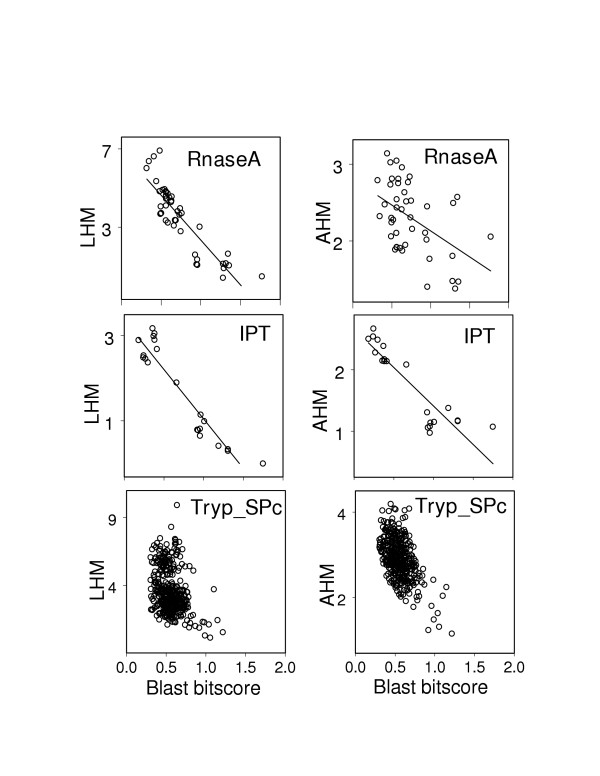
Hausdorff measure (in Angstroms) for loop (LHM) and aligned (AHM) regions is plotted versus the normalized Blast bitscore for three families: Pancreatic ribonucleases (RnaseA), Ig-like plexins/transcription factors (IPT) and Trypsin-like serine proteases (Tryp_SPc). Solid line shows the linear regression fit of the data.

As was shown previously, the evolutionary relatedness between proteins can be successfully gauged from the comparison of their loop regions [7]. Indeed, Table 2 and Figure 1 show that within the families of homologous proteins, the structural changes in loops are strongly coupled with evolutionary distance, which in the first approximation can be estimated using normalized Blast score. The structural-sequence dependence in loop regions for 71% of our protein families can be well described by a linear model and for 88% of protein families the linear correlation coefficients are found to be statistically significant. Comparing different measures of structural similarity one can see that AHM performs somewhat better than other quantities yielding 90% of families with statistically significant linear correlation coefficients (with P-value < 0.01) and 80% of families with *r*^2 ^> 0.9.

**Table 2:** Table shows the median of Pearson correlation coefficients, fraction of families with statistically significant correlation (P-value less than 0.01) and the fraction of families with the ratio *r*^2 ^higher than 0.9 for each measure of structural similarity used in the study.

	***Median correlation coefficient***	***% families with significant correlation***	***% families with r^2 ^> 0.9***
***RMSD***	-0.81	90	71
***AHM***	-0.82	90	80
***LHM***	-0.76	88	71

However, not all families exhibit such good correlation. One example of a protein family showing particularly low LHM correlation is the family of Actin depolymerisation factor/cofilin-like domains (ADF). The sequence-structure correlation for loop regions of this family is not statistically significant (the Pearson correlation coefficient is close to zero) whereas the sequence-structure correlation for the protein core is very high (ρ = -0.85 with AHM). Indeed, different proteins of this family show distinctly different loop conformations and evolutionary analysis of ADF family argued that the insertions present in the vertebrate ADF/cofilins (and not present in non-vertebrate cofilins) might be important for nuclear function of mammalian cofilins [22]. Therefore, in this case the structural heterogeneity of loop regions can be explained by the acquisition of a new distinct function by some members of this family. For some families, for example, Trypsin-like serine protease (Tryp_SPc), neither LHM (ρ = -0.31) nor AHM (ρ = -0.55) similarity measures exhibit a good sequence-structure correlation (Figure 1(c)).

Among families with particularly high LHM correlation are the families of Xylose isomerase (Xylose_isom), Class I Histocompatibility antigen (domains alpha 1 and 2, MHC_I), Protein tyrosine phosphatase (PTPc) and others. Figure 1 shows two families with high sequence-structure correlation using the LHM measure: Ig-like plexins (IPT) and Ribonucleases A (RnaseA). The IPT family is characterized by high sequence-structure correlation for both core (ρ_AHM _= -0.90) and loop regions (ρ_LHM _= -0.94). On the other hand, the protein core structure of the RnaseA family changes very little with sequence whereas the loop structure gradually diverges as sequence becomes more and more dissimilar (ρ_AHM _= -0.48, ρ_LHM _= -0.87).

**Figure 2:**
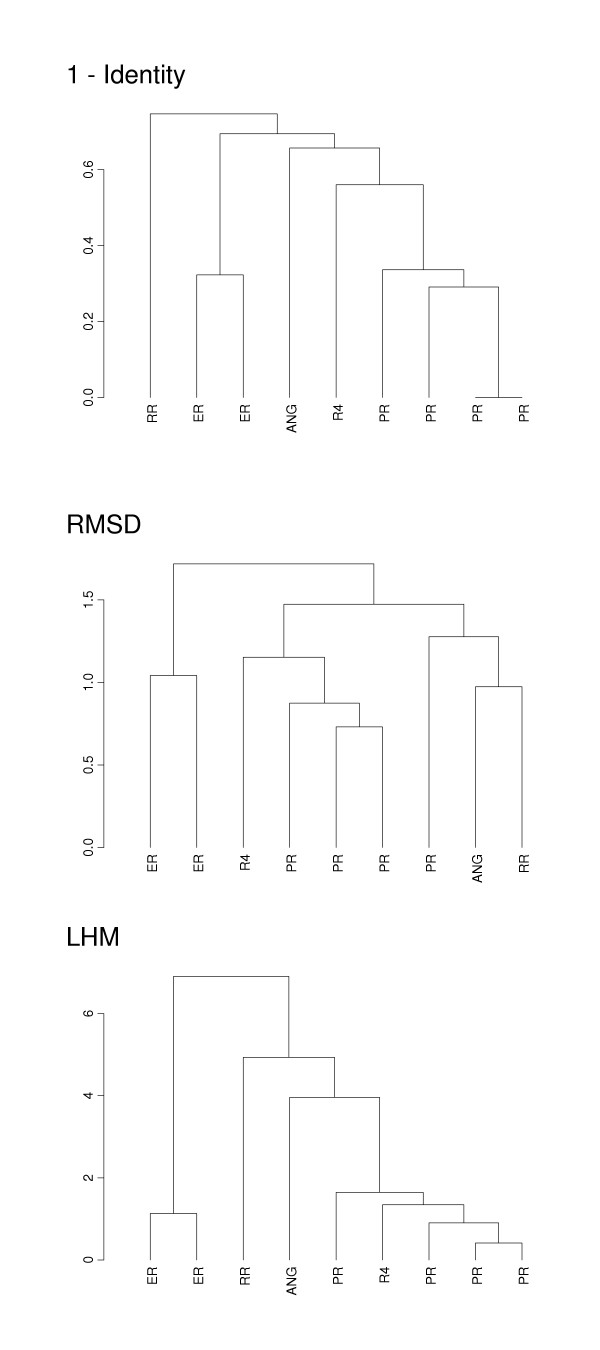
Complete linkage cluster tree produced using fraction of non-identical residues (p-distance), RMSD (Å), and LHM (Å) is plotted between proteins from Pancreatic ribonuclease family (RnaseA). Five major groups of RnaseA family according to Rosenberg et al [23] are: eosinophil ribonucleases (ER), pancreatic ribonucleases (PR), angiogenins (ANG), Rana ribonucleases (RR) and ribonuclease 4 (R4). The maximum parsimony tree described by Rosenberg et al [23] is given in the Phylip format: (RR, ((ANG, R4), (PR, ER))).

To understand whether significant sequence-structure correlation for loop regions has an underlying biological meaning, we performed a cluster analysis of proteins from two diverse families, Ribonuclease A (RnaseA), and SH2 domain (SH2, ρ_AHM _= -0.48, ρ_LHM _= -0.78), using different measures of sequence and structural similarity. Figure 2 depicts the cluster trees constructed using distance/similarity matrices which were based on the fraction of non-identical residues (p-distance), RMSD and LHM for these two families.

The RnaseA family represents a very interesting example to study as it is characterized by considerably different catalytic efficiency and substrate preferences among family members and the different aspects of its activity is not well understood. Although cysteines that form disulfide bonds, catalytic histidines and lysine residues are mostly structurally and sequence conserved, there is a great variability in sequence between other regions of RnaseA proteins [23,24]. We compared the obtained cluster trees (Figure 2) with the maximum-parsimony phylogenetic tree derived by Rosenberg et al [23], the Phylip format of this tree is given in the captions of Figure 2. As shown in this figure, the RMSD-based tree divides pancreatic ribonucleases (PR) into two groups and puts together two very different proteins: angiogenin (ANG) and *Rana *ribonuclease (RR) although angiogenin has a very weak enzymatic activity and is a tumor-growth promoter while *Rana *ribonuclease P-30 has ribonuclease activity and antitumor effects. In contrast to the RMSD cluster tree, distance matrices based on the loop (dis)similarity measure correctly cluster the representatives of the five major groups of the Ribonuclease family as per Rosenberg et al [23]. Although the topology of the p-distance based cluster tree is somewhat different from the topology of the LHM based tree (with bootstrap support less than 0.001), it also produces a biologically meaningful clustering as judged from Rosenberg et al [23].

**Figure 3:**
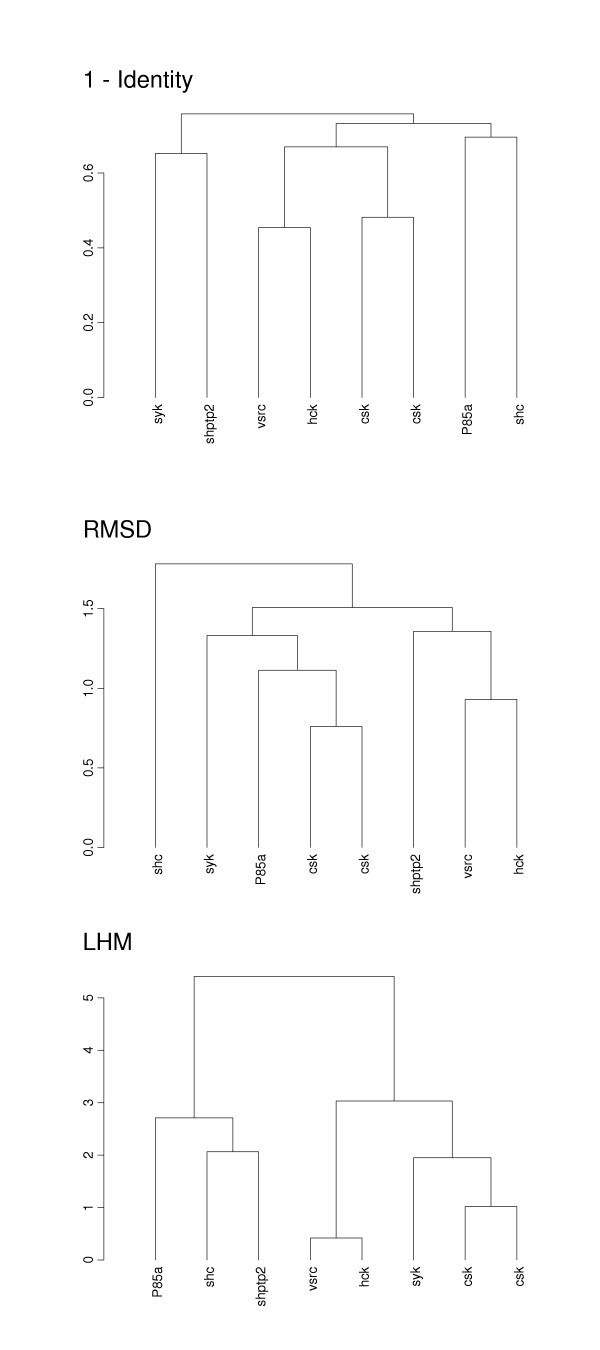
Complete linkage cluster tree produced using fraction of non-identical residues (p-distance), RMSD (Å), and LHM (Å) is plotted between proteins from SH2 family (SH2). The classifications of SH2 domains according to [25, 26] are given in the parentheses: syk (1B, B), shptp2 (4, C), vsrc (1A, A), hck (1A, A), csk (1B, B), P85a (3, D) and shc (4,).

SH2 domains represent phosphor-tyrosyl peptide binding modules which are found in many signaling proteins. The specificity of phosphate interaction with a protein has been attributed to the hydrophobic pocket which is mostly formed by two loop regions [25]. Our analysis shows that indeed the loop regions have a much higher accuracy in clustering of functional subfamilies of SH2 domains. Comparing our cluster trees with the classification of Songyang et al [26] and cluster trees of SH2 phosphotyrosyl binding sites [25] we can see from Figure 3 that p-distance based and RMSD based distance matrices cluster correctly two representatives of the "1A" subfamily (vsrc, hck), but separate proteins from subfamily "1B" (csk, csk, syk) and "4" (shptp2 and shc). In contrast, these subfamilies ("1B" and "4" [26]) are very well supported by the cluster tree which is based on the LHM measure. The bootstrap calculations (see Methods) show that the LHM based topology is supported by the p-distance based clustering algorithm at less than the 0.001 level. Different partitions of this tree are supported at higher but still non-significant levels, namely 0.11 for the "1B" subfamily (csk, csk, syk) and 0.01 for the subfamily "4" (shptp2 and shc). This in turn indicates that the two cluster trees can be considered statistically different.

## Conclusions

Here we have presented an analysis of how the structure of protein loops changes in evolution as homologous proteins diverge from each other. We showed that for the majority of protein families there exists a statistically significant linear correlation between measures of sequence similarity and average loop structural similarity. This in turn suggests that loops change in evolution via a stepwise insertion or deletion process and clearly one can not portray even longer loop regions as "irregular conformations" or "random coils". Indeed, our results imply that, in general, loops are under constant evolutionary constraints which, apparently, are weaker than those for a protein core but still strong enough to preserve the loop overall structure. Since loops do not contribute much to the protein core stability, these constraints predominantly arise from the importance of loops in interacting with ligands, other proteins and cells, as well as a possible role of loops in protein folding.

Modeling of insertion and deletion events in evolution poses a lot of difficulties and protein evolution is usually reconstructed based only on the aligned regions of proteins. We demonstrated that loop regions which usually correspond to the non-aligned protein regions can be very important in inferring the phylogenetic history of a protein family. Moreover, it was shown, that sometimes sequence and structure similarity measures comparing proteins in their core are not sensitive enough to detect subtle (dis)similarities between the subfamilies. Loop-based measures which emphasize the dissimilarities between different protein members can shed light on the evolutionary relationships between homologous proteins.

## Authors' contributions

AP and TM contributed equally to this paper.
